# Editorial: Coaches’ role in youth sports performance: early specialization *versus* long-term development

**DOI:** 10.3389/fphys.2024.1370588

**Published:** 2024-02-09

**Authors:** Daniel A. Marinho, Maria do Socorro Cirilo de Sousa, Ricardo Ferraz, Henrique P. Neiva

**Affiliations:** ^1^ Department of Sports Sciences, University of Beira Interior, Covilhã, Portugal; ^2^ Physical Education Department, Regional University of Cariri, Crato, Brazil

**Keywords:** sports performance, youth specialization, youth sports, long-term development, coaching philosophy

The Research Topic *Coaches*’ *Role in Youth Sports Performance: Early Specialization* Versus *Long-Term Development* aimed to explore the complex role that coaches play in the growth and performance of young athletes. This research holds particular significance in the ongoing discussion about the dual considerations of early specialization and the comprehensive framework of long-term athletic development. The articles in this Research Topic show the impact of some training factors on the delicate balance between guiding young athletes towards specialization and ensuring their sustained and well-rounded athletic development in the long term.

As highlighted by Zhang et al., coaches play a crucial role in improving motor performance, emphasizing the importance of increasing elasticity, which plays a key role in the long-term development process of young athletes. The authors considered that the stretch reflex and other involuntary functions of the nervous system are key in enhance the stretch-shortening cycle (SSC). Moreover, the authors noted that coaches emphasizing eccentric pre-stretching during plyometric training (PT) can enhance athletes’ muscle activation and that PT appears valuable for improving players’ balance. However, important factors such as age, gender, skill level, and intervention duration do not seem to have significant effects on PT. In conclusion, coaches are encouraged to strategically incorporate PT into soccer players’ overall training programs, aligning it with various training approaches. This includes incorporating various PT forms like drop jumps, countermovement jumps, squat jumps, alternate leg bounding, hopping, and more. This information offers coaches various options to adapt workouts for specific goals. Strength and conditioning professionals can judiciously employ PT, either independently or in combination with other methods, to optimize training outcomes.

Furthermore, Hernández-Martinez et al. conducted a pilot investigation clarifying the crucial role of the coach in effective warm-up strategies for young male football players. Despite observing modest gains in running and kicking speed following warm-ups that incorporated static and ballistic stretching, the study highlights the coach’s responsibility in choosing warm-up exercises that meet players’ specific needs. This research highlights the role of the coach in warm-up decisions and serves as a valuable resource for optimizing training strategies, which can be helpful in long-term development. In conclusion, this study provides practical knowledge for coaches working with young male football players, helping them to improve performance in their daily responsibilities.

Furthermore, Hermassi et al. explored how coaches play a crucial role in fostering overall development according the physical and academic performance of young athletes. This study highlighted the significant impact of biological maturity on physical abilities, revealing distinct patterns of development in agility across different age groups. The study positions coaches not only as promoters of overall development but also emphasizes the interconnection between physical and academic aspects in a young athlete’s journey. Coaches with this insight can play a crucial role in the overall development of athletes whose success transcends the sports field and extends into academic domains.

Additionally, Soares et al. conducted a significant investigation into early specialization in basketball, highlighting the essential role of coaches in guiding young players towards optimal physical, technical, and tactical development. The findings are particularly useful for coaches, showing that improvements in basketball-specific performance during a competitive season were more visible among female players, while male players maintained their physical fitness levels. This emphasizes the need for differentiated guidance from coaches based on gender, age, and estimated maturation status. The study also investigates the growth characteristics of Brazilian adolescent basketball players, reflecting broader trends and cautioning against rushed conclusions from somatic indicators. It underscores the coach’s crucial role in understanding and interpreting maturation, urging caution about generalizations based on limited validity in prediction equations. In conclusion, this study serves as a guide for coaches, young athletes, and club stakeholders, advocating for a balanced approach in setting expectations for season-long physical fitness improvements among young basketball players. With this information, coaches could make thoughtful decisions that positively impact the overall development of their players.

Lastly, Li et al. examined the training experiences of elite Chinese youth football players, highlighting the crucial role of coaches in their development. This study explored the connections between early specialization, training volume, maturity status, and musculoskeletal injuries, emphasizing the coach’s vital role in ensuring the wellbeing of young talents. The results suggested a correlation between early specialization and a heightened risk of reporting injuries, particularly acute ones, with ankle injuries being prevalent. The coach is considered essential in addressing the root causes, such as inadequate training, insufficient physical readiness, and restricted athletic abilities resulting from early specialization. To mitigate injury risks, the study recommends coaches explore alternative approaches, such as early diversification. Coaches are responsible for creating an environment that supports the holistic development of young players, including balanced exercise, strength training, and health monitoring. This investigation highlights the coach’s responsibility in promoting balanced exercise, adequate rest, and consistent assessments to ensure the wellbeing of elite youth football players. As coaches implement injury prevention strategies, they play a significant role in fostering the overall health and longevity of young football players pursuing elite paths.

In summary, the debate over early specialization *versus* long-term development in sports underscores multifaceted factors that impact athletic excellence. Coaches, as central decision-makers, face the challenge of choosing the most effective approach, requiring a deep understanding of physiological, psychological, and developmental aspects. Beyond promoting physical skills, coaches significantly influence both the physical and academic dimensions of a young athlete’s journey. Ultimately, coaches hold the key to think about the complex dynamic of specialization, making informed decisions that positively impact the overall development and wellbeing of young athletes on the path to elite success. Therefore, achieving overall growth and wellbeing requires a capable balance, considering the diverse landscape of youth sports and recognizing individual needs and developmental trajectories.

